# Human Papillomavirus types distribution among women with cervical preneoplastic, lesions and cancer in Luanda, Angola

**DOI:** 10.11604/pamj.2016.24.268.9678

**Published:** 2016-07-22

**Authors:** Paciência de Almeida Damião, Michelle Oliveira-Silva, Miguel Ângelo Moreira, Natalia Poliakova, Maria Emilia RT de Lima, José Chiovo, Alcina Frederica Nicol

**Affiliations:** 1 Departamento de Anatomia Patológica do Hospital Militar Principal, Instituto Superior, Luanda, Angola; 2 Oswaldo Cruz Institute, Fiocruz, Rio de Janeiro, Brazil; 3 Division of Genetic, National Institute of Cancer, Rio de Janeiro, Brazil; 4 Laboratory of Interdisciplinary Medical Research, LIPMED, IOC, Fiocruz, Rio de Janeiro, Brazil

**Keywords:** HPV, cervical lesions, Angola, Africa

## Abstract

**Introduction:**

Cervical cancer is the leading cause of cancer deaths among females in Angola and human papillomavirus (HPV) is the main risk factor for the development of pre-cancerous squamous intraepithelial lesions. The diversity and frequency of HPV types in Angola has yet to be reported.

**Aim:**

To determine the frequency of HPV among women with squamous intraepithelial lesions from women in Luanda, Angola.

**Methods:**

Study participants included women diagnosed with cytological abnormalities that voluntarily provided Pap smears (n = 64). Genomic DNA was extracted from the samples for use as templates in the PCR amplification of HPV sequences. PCR products were sequenced to determine HPV type.

**Results:**

HPV DNA was detected in 71.9% (46/64) in the samples. A higher diversity of HPV types was found in the cytological lesions, such as ASCUS and LSIL (HPV16, 6, 18, 31, 58, 66, 70 and 82, in order of frequency) than that detected for HSIL and SSC (HPV16, 18, 6 and 33). The most prevalent HPV type were: HPV16, HPV6 and HPV18.

**Conclusion:**

This is the first report on HPV type diversity and frequency in woman of Angola. The results suggest that large-scale studies across Africa would improve our understanding of interrelationship between HPV infections and cervical cancer. More directly, the identification of the HPV types most prevalent suggests that women in Angola would benefit from currently available HPV vaccines.

## Introduction

Human Papillomavirus (HPV), a common sexually transmitted infection, is the major risk factor for squamous intraepithelial lesions (SIL) and cervical cancer [1]. Cervical cancer is the third most common cancer among women worldwide and the second highest in the developing countries [2]. Annually, 530,000 new cases of cervical cancer and 275,000 deaths caused by cervical cancer are reported, and more than 85% of these cases and deaths occur in developing countries. According to the World Health Organization the incidence rate of cervical cancer in Angola is age-standardized incidence rate of 35.5 per 100,000. It is estimated that there are 2,072 new cases of cervical cancer each year and that 1,141 women die of this cancer annually in Angola [3]. The carcinogenic HPV genotypes have been recognized as the causative agent of cervical cancer, associated with persistence and progression [2], a comprehensive data on HPV genotype prevalence and distribution in Middle region of Africa is still lacking [4]. Furthermore, a wide diversity of HPV types was reported for other regions [5–13]. However there is a paucity report in Angola. Thus, the prevalence and distribution of HPV genotypes in Africa among women is required moreover the study of HPV type frequency is important to further development of screening tests and prevention through the use of HPV vaccination. Therefore, the aim of this study was to determine the HPV diversity types in cervical pap smears with cytological abnormalities from women attending in Hospital Militar Principal in Luanda, Angola. To our knowledge, this is the first study to analyze the frequency of HPV types among women with neoplastic cervical lesions and cancer in Angola.

## Methods

The present study had a cross-sectional design and consisted of 64 women diagnosed with cytological cervical abnormalities, aged from 20 to 65 years. The samples of exfoliated cervical cells were collected from the Laboratory of Pathology in Hospital Militar Principal, Luanda city, between 2006 to 2008. Interpretation of cytological specimens was reported according to the Bethesda System 2001 [14]. Out of 64 women included in this study, 13 (20.3%) had the cytology classified as atypical squamous cells of undetermined significance (ASCUS), 29 (45.3%) were low grade squamous intraepithelial lesion (LSIL), 16 (25.0%) were high grade (HSIL), and 6 (9.4%) had squamous cell carcinoma (SCC). The Pap smear slides were maintained in recipient containing xylene 100% until removal of the coverslip. Subsequently, the smear was scrapped with a sterile scalpel from the slide to a polypropylene tube of 1.5 mL. DNA was isolated from this material using the Invisorb Spin Forensic Kit (Invitek, Ca-USA) following the manufacturer's instructions but with slight modification in elution step by using 30 µL of elution buffer. The quality of the DNA isolated was evaluated using the NanoDrop^®^ ND-1000 spectrophotometer and by the amplification of a fragment of ß-globin gene. HPV detection was carried out by nested-PCR system, using in the first round the MY09/1 and in the second round GP5+/6+ primers. The amplicons were purified with the Illustra GFX PCR and Gel Band Purification Kit (GE Healthcare, Buckinghamshire, UK) before being submitted to direct sequencing using Big Dye Terminator Kit (Applied Biosystems -AB Applied Biosystems, CA-USA) in a ABI3730 sequencer at the Genomic DNA Sequencing Platform (PDTIS) of Fiocruz. The specimens that could not be typed by direct sequencing were cloned with TOPO TA Cloning Kit (Invitrogen, Carlsbad, CA) and 12 clones from each sample were submitted to sequencing. Identification of HPV types was carried out with phylogenetic analysis using the MEGA 4.0, Neighbor-Joining method, Kimura's-2-Parameter distance model. The strength of each node was evaluated by bootstrap with 1.000 replicates. Correlation between HPV types found and cytology results was assessed using Fisher's exact test.

**Ethics:** The Hospital Militar Principal, Luanda, Angola and the Institutional Review Board (IRB) from Oswaldo Cruz Foundation (Fiocruz), Rio de Janeiro, Brazil under the protocol nº 526/09 approved this study.

**Statistical Analysis:** Data were analyzed by means of the STATA/SE 10.1 software, with the non-parametrical tests of Kruskall-Wallis, Student's t, Fisher's exact and chi-square. P-value <0.05 was considered statistically significant.

## Results

**Figure 1:**
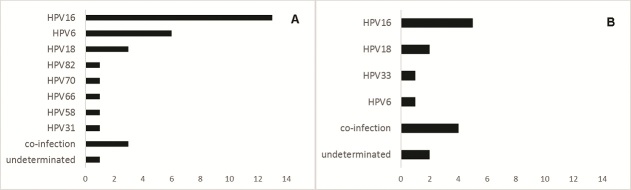
The most common HPV types by disease severity

Of the 64 samples included in this study, 59 (92.2%) had the amplified DNA for ß-globin primers and 46 (71.9%) of these were positive for HPV DNA. The presence of HPV according to the cytological lesions was 53.8% (7/13) for ASCUS, 82.8% (24/29) for LSIL, 68.8% (11/16) for HSIL and 66.7% (4/6) for SCC. Electropherograms that presented overlapping peaks suspected of co-infection with different HPV strains were found for 10 patients which had the amplification product by PCR subjected to molecular cloning. Moreover, the HPV types for three positive cases (2 of HSIL and 1 of LSIL) could not be identified by sequencing and therefore classified as undetermined type. All 50 HPV sequences identified in this study were submitted to GenBank. Phylogenetic analysis showed a total of 37 single infections and 6 cases of different HPV types in co-infection. Low-risk HPV types HPV6 and 70 were found on single infection in 8 women diagnosed with ASCUS (1), LSIL (6) and HSIL (1). High-risk HPV types were found in other 29 women with single infections and in 6 women with multiple infections by two different types of HPV (Table 1). Within the 37 cases of single infections, the prevalent HPV type found was HPV16 (51.4%; 19/37), followed by HPV6 (18.9%; 7/37), HPV18 (13.5%; 5/37), and one each for HPV types HPV31, 33, 58, 66, 70 and 82 (2.7%; 1/37). Among the six cases of multiple infections, four showed co-infection by both high-risk types (two cases with HPV66/53) and other two cases by HPV16/53 and HPV16/58. Furthermore, two women showed co-infection with low-risk and high-risk types as HPV6/35 and HPV6/66. These findings about the HPV typing were showed according of cytological results in Table 1. No statistical significance was found between the HPV types and the Pap smear diagnostic by Fisher´s exact test. In categorized analysis by disease severity according to type of cytological lesions grouped as ASCUS plus LSIL and HSIL plus SSC, we observed a higher diversity of HPV types in the first group (Figure 1,A) than in the group that includes women diagnosed with more severe disease (Figure 1,B). The HPV16, 6, 18, 31, 58, 66, 70 and 82 were found in less severe pathological disorders while HPV16, 18, 6 and 33 were identified to more severe illnesses.

**Table 1:** HPV type across cervical cytological abnormalities

HPV typing			Pap smear diagnostic			
			ASCUS(N=7)	LSIL (N=24)	HSIL(N=11)	SCC (N=4)
**Single infections**	High-risk	HPV16	4(57.1%)	10(41.7%)	3 (27.3%)	2 (50%)
		HPV18	1	2 (8.3%)		2 (50%)
		Others		4 (16.7%)	1 (9.1%)	
	Low-risk	HPV6	1(14.3%)	5 (20.8%)	1(9.1%)	
		HPV70			1 (4.2%)	
**Multiple infections**		HPV16/58	1 (14.3%)			
		HPV16/53			1(9.1%)	
		HPV53/66		1 (4.2%)	1(9.1%)	
		HPV6/35			1(9.1%)	
		HPV6/66	1(9.1%)			
**Uncharacterized types**			1(4.2%)	2(18.2%)		

## Discussion

To the best of our knowledge, this is the first report on diversity of HPV types in Angola, Middle Africa region. Variations in the geographical distribution of HPV genotypes in women with cervical pathology have been reported in sub-Saharan Africa. Although this study included samples that are not representative of general population of Angola, the higher frequency HPV types found in this study, HPV16 and HPV18, does not differ from those described in developed countries and other African countries [4, 15] as Ethiopia [16], Ghana [17], Uganda [13], Algeria [5], Marroco [17], South Africa [18] and Mozambique [19]. However, this higher frequency of HPV16 here found is different than previous reports in other African countries that found as the most prevalent HPV type the HPV58 in Botswana [10], HPV45 in Cameroon [20], HPV53 in Gabon [21], HPV35 in Burkina Faso [22], HPV52 in Kenya [7]. Moreover, previous studies performed in countries geographically near to Angola show a different prevalence of HPV types. In Guinea, the four most prevalent HPV type were HPV16, 33, 58 and 18, while in border countries with Angola, as Democratic Republic of the Congo which 85.5% of the women studied were HIV-seropositive, the most prevalent HPV type were HPV68, 35, 51 and 52 [23] and in Zambia that all patients were HIV-seropositive, the types found were HPV52, 58, 53 and 16 [24]. On the other hand, the higher frequency of HPV types 16, 18,31, 58, 66 and 33 here identified in Angola is similar to recent data of a meta-analysis on Africa continent in women with normal cytology which was reported a prevalence of HPV16, followed by 52, 18, 31, 35, 45, 58 and 66 [3]. A stratification of specific HPV type prevalences by disease severity contributes on identification of appropriate tools for HPV typing and assessing the future impact of HPV vacines [15]. Using this approach, we confirmed that all our cases of SCC in Angola were positive for HPV16 or HPV18 in agreement with the ~70% global estimate found by other meta-analyses [4, 15, 25–27]. The present study had some limitations, such as the relatively small sample size among the different lesions stage, limiting subgroup analyses. However, our study highlighted important and new data regarding the main HPV types in Luanda-Angola. All these data contributes to the understanding of the diversity and distribution of HPV types in the African continent and points to the challenge in understanding of interrelationship between HPV infections and cervical cancer and the need for large-scale studies in Africa. Moreover the high prevalence of HPV types found in bivalent, quadrivalent and nonavalent vaccines demonstrate that this woman will greatly benefit from current HPV vaccines.

## Conclusion

The present study provides the first report on the frequency and types of HPV in women of Angola and contributes to the understanding of the diversity and distribution of HPV types in Africa.

### What is known about this topic

- High risk HPV type is the main causative agent of cervical cancer, associated with persistence and progression;
- There are Variations in the geographical distribution of HPV genotypes in Africa;
- The diversity and frequency of HPV types in Angola has yet to be reported. Over 1,141 women die of cervical cancer annually in Angola.

### What this study adds

- The most prevalente HPV types were HPV16, HPV6 and HPV 18;
- Woman on this region will greatly benefit from current HPV vaccines;
- A higher diversity of HPV types was found in the cytological abnormal lesions. This is the first report on HPV type diversity and frequency in woman of Angola.
